# Success and time implications of SpO_2_ measurement through pulse oximetry among hospitalised children in rural Bangladesh: Variability by various device-, provider- and patient-related factors

**DOI:** 10.7189/jogh.12.04036

**Published:** 2022-04-23

**Authors:** Ahmed Ehsanur Rahman, Shafiqul Ameen, Aniqa Tasnim Hossain, Sabrina Jabeen, Tamanna Majid, Azim Uddin AFM, Tania Sultana Tanwi, Goutom Banik, Md Ziaul Haque Shaikh, Md Jahurul Islam, Sabina Ashrafee, Husam Muhammad Shah Alam, Ashfia Saberin, Ehtesham Kabir ANM, Sabbir Ahmed, Mahbuba Khan, Anisuddin Ahmed, Qazi Sadeq-ur Rahman, Mohammod Jobayer Chisti, Steve Cunningham, Muhammad Shariful Islam, David H Dockrell, Harish Nair, Shams El Arifeen, Harry Campbell

**Affiliations:** 1 NIHR Global Health Research Unit on Respiratory Health (RESPIRE), Usher Institute, The University of Edinburgh; 2 Maternal and Child Health Division, icddr,b (International Centre for Diarrhoeal Disease Research, Bangladesh), Dhaka, Bangladesh; 3 Directorate General of Health Services, Ministry of Health and Family Welfare, Government of Bangladesh; 4 Save The Children, Dhaka, Bangladesh; 5 World Health Organization, Dhaka, Bangladesh

## Abstract

**Background:**

Hypoxaemia is one of the strongest predictors of mortality among children with pneumonia. It can be identified through pulse oximetry instantaneously, which is a non-invasive procedure but can be influenced by factors related to the specific measuring device, health provider and patient. Following WHO's global recommendation in 2014, Bangladesh decided to introduce pulse oximetry in paediatric outpatient services, ie, the Integrated Management of Childhood Illness (IMCI) services in 2019. A national committee updated the existing IMCI implementation package and decided to test it by assessing the pulse oximetry performance of different types of assessors in real-life inpatient settings.

**Methods:**

We adopted an observational design and conducted a technology assessment among children admitted to a rural district hospital. Eleven nurses and seven paramedics received one-day training on pulse oximetry as assessors. Each assessor performed at least 30 pulse oximetry measurements on children with two types of handheld devices. The primary outcome of interest was obtaining a successful measurement of SpO2, defined as observing a stable (±1%) reading for at least 10 seconds. Performance time, ie, time taken to obtain a successful measurement of SpO_2_ was considered the secondary outcome of interest. In addition, we used Generalized Estimating Equation to assess the effect of different factors on the pulse oximetry performance.

**Results:**

The assessors obtained successful measurements of SpO_2_ in all attempts (n = 1478) except one. The median time taken was 30 (interquartile range (IQR) = 22-42) seconds, and within 60 seconds, 92% of attempts were successful. The odds of obtaining a successful measurement within 60 seconds were 7.3 (95% confidence interval (CI) = 3.7-14.2) times higher with a Masimo device than a Lifebox device. Similarly, assessors aged >25 years were 4.8 (95% CI = 1.2, 18.6) times more likely to obtain a successful measurement within 60 seconds. The odds of obtaining a successful measurement was 2.6 (95% CI = 1.6, 4.2) times higher among children aged 12-59 months compared to 2-11 months.

**Conclusions:**

Our study indicated that assessors could achieve the necessary skills to perform pulse oximetry successfully in real-life inpatient settings through a short training module, with some effect of device-, provider- and patient-related factors. The National IMCI Programme of Bangladesh can use these findings for finalising the national IMCI training modules and implementation package incorporating the recommendation of using pulse oximetry for childhood pneumonia assessment.

Each year, approximately 24 000 children under-five years of age die due to pneumonia in Bangladesh, making it the major killer among this age group [1,2]. In addition to mortality, the morbidity-burden associated with childhood pneumonia is also remarkably high in Bangladesh as it causes around 4 million episodes of illness every year, of which around one million are severe episodes, and approximately half a million require hospitalisations [3,4]. Many other low- and middle-income countries (LMICs) suffer from a similarly high burden of childhood pneumonia, where the majority of the deaths can be prevented by appropriate management and immediate treatment [3]. Hypoxaemia, defined as low (<90%) oxygen saturation (SpO_2_) in arterial blood, is common among children with pneumonia and is regarded as one of the strongest predictors of adverse clinical outcomes, including treatment failure and mortality [5-8]. Early identification of hypoxaemia and prompt management with oxygen therapy can significantly avert deaths due to pneumonia [9,10]. Thus, strengthening the health system for early identification and management of hypoxaemia can play a crucial role in achieving the Sustainable Development Goals (SDG) target of reducing under-five mortality to 25 per thousand live births or below by 2030 and the Integrated Global Action Plan for Pneumonia and Diarrhoea (GAPPD) target of reducing pneumonia specific under-five mortality rate to 3 per thousand live births or below by 2025 [11-13].

Pulse oximetry is an indirect, simple and non-invasive procedure to measure SpO_2_ using a pulse oximetry device and identify hypoxaemia instantaneously [14]. Initially, its use was limited to monitoring SpO_2_ during anaesthesia and almost exclusively in resource-intensive settings such as Intensive Care Units (ICUs) [15-18]. However, the size and the cost of pulse oximetry devices have significantly decreased due to technological advancements in the last couple of decades [19,20]. Hence, pulse oximetry has become the standard of care in outpatient, emergency and inpatient settings, mainly in high-income countries. Due to the high burden of hypoxaemia among children with pneumonia and the potential impact of introducing pulse oximetry on child survival, the World Health Organization (WHO) has recommended introducing pulse oximetry in paediatric outpatient services, ie, Integrated Management of Childhood Illness (IMCI) services since 2014 [4,20-22]. In 2019, the National IMCI Programme of the Government of Bangladesh decided to introduce pulse oximetry in routine IMCI services to identify hypoxaemia among children presenting with cough and difficulty breathing [23,24]. The National IMCI programme formed a high-level committee with IMCI experts to integrate the recommendation of using pulse oximetry in the National IMCI training modules and implementation package [24]. The committee incorporated a short (one-day) module on pulse oximetry into the existing IMCI training package [24].

Various factors influence pulse oximetry performance, particularly related to obtaining successful measurements and the time required for that [25-30]. These factors can be divided into three broad categories: device-related factors, patient-related factors, and provider-related factors. Unfortunately, most of the evidence in this regard is from resource-rich environments and high-income country settings and is predominantly based on qualitative explorations [31-36]. There is a dearth of quantifiable evidence regarding the success and time implications for performing pulse oximetry and their variability by various factors in resource-limited settings, particularly in the context of Bangladesh. Hence, the National IMCI Programme of Bangladesh decided to test the newly developed training module in a paediatric inpatient setting and assess the pulse oximetry performance of different types of assessors by different types of devices before finalising the training modules and implementation package and feasibility assessment through demonstration in outpatient-based routine IMCI services [24]. In this paper, we present key findings from the pre-implementation testing, which guided programme planning, and implementation in real-life settings.

## METHODS

### Study design

We conducted a technology assessment adopting an observational design, where study recruited assessors received training based on the updated module on pulse oximetry and used handheld pulse oximetry devices to assess SpO_2_ status among children hospitalised in a rural district hospital in Bangladesh.

### Study settings

The study was conducted in Kushtia district, which is situated approximately 200 km west of Dhaka city, the capital of Bangladesh. The Government of Bangladesh decided to conduct this study and subsequent feasibility assessment through field demonstration in this district as the under-five mortality rate of Kushtia was somewhat similar to the average under-five mortality rate in Bangladesh [37]. We conducted the assessments in the paediatric inpatient unit of Kushtia District Hospital, which is the highest-level referral facility (secondary level with 250 inpatient beds) in the Kushtia district. The paediatric inpatient unit of Kushtia District Hospital provides inpatient services to approximately 3000 under-five children per year. The majority of the admissions are due to pneumonia, sepsis, prematurity related complications, and perinatal birth asphyxia. Pulse oximetry was not routinely used in Kushtia District Hospital at the time of this study.

### Study participants

We included children aged 2-59 months who were admitted to the paediatric inpatient unit and whose parents consented to participate in the study. Exclusion criteria included unconsciousness, active bleeding and drowning at the time of assessment as they required urgent medical interventions, which were not directly dependent on SpO_2_ assessment. In government settings, routine IMCI services are primarily provided by trained nurses and paramedics. Hence, we recruited study nurses and paramedics (graduates from Medical Assistant Training School) who received a one-day long training based on the newly developed IMCI training module on pulse oximetry. The training included the basic pathophysiology of hypoxaemia, the function of a pulse oximetry device and its parts, steps of performing pulse oximetry and instructions for the maintenance of the device.

### Sample size and sampling

The primary outcome of interest was obtaining a successful measurement of SpO_2_ through pulse oximetry. Since we did not have any previous estimate from Bangladesh, we considered the maximum variance, ie, a success rate of 50%. In order to address the cluster effect (each assessor as a cluster), we adjusted the sample size with a design effect of 1.25. We also considered a non-response rate of 10%. The final adjusted sample size was 534 eligible children. We employed 11 nurses and seven paramedics as assessors. We wanted to enrol an equal number of participants per assessor. Hence, each assessor was responsible for performing at least 30 pulse oximetry assessments among hospitalised children. We adopted a time sampling approach as all children admitted to the inpatient department were approached and enrolled during the data collection period based on the inclusion and exclusion criteria. It took around 2-3 days per assessor to achieve the required target. On the day the required sample size (minimum of 30 children) was reached for each assessor, they continued enrolment and data collection until the end of their shifts.

### Data collection methods and procedures

We collected data between 29 October 2020 to 03 April 2021. Data collection methods included data extraction from routine hospital records, structured observation of pulse oximetry by study nurses and paramedics, and structured survey of the study nurses and paramedics on the challenges of using pulse oximetry on hospitalised children. Information regarding the children's basic demographic and clinical characteristics, such as age, sex, weight and diagnosis at admission, was extracted from hospital inpatient case recording forms using a structured form. The assessors performed pulse oximetry on each child immediately after admission using two types of handheld devices (ie, Lifebox AH-M1, and Masimo Rad-5V) with adequately fitted paediatric probes. The national committee selected the pulse oximetry devices based on technical specifications and in consultation with technical experts [23]. Additional technical details and specifications regarding pulse oximetry devices are summarised in Table S1 in the **Online Supplementary Document**. Independent observers assessed the pulse oximetry performance of study nurses and paramedics using a structured observation tool. A measurement was considered successful if the signal strength was shown adequate (four or more out of five) and a stable (±1%) SpO_2_ reading was observed for at least 10 seconds. A measurement was considered unsuccessful if the signal strength was inadequate and they could not establish a stable SpO_2_ reading within three minutes. Performance time was calculated from placing the probe onto the fingertip to obtaining a successful measurement using a digital stopwatch. After completion of the structured observation, the assessors completed a self-administered survey tool on the challenges of performing pulse oximetry on hospitalised children. The self-administered survey tool consisted of five-scale Likert scale statements with the following options: very easy, easy, neither easy nor challenging, challenging and very challenging. A medical graduate with prior experience in using pulse oximetry devices received IMCI training and monitored the data collection process as an on-site clinical supervisor. These supervisors provided feedback to the data collectors to improve the data quality. No additional refresher training was organised for the assessors during the data collection process in order to follow the exact procedure that applies for the government-appointed IMCI service providers.

### Data analysis

We used the statistical software package Stata version 14.0 for data analysis [38].

Descriptive statistics (frequencies and percentages) were used to present the basic demographic and clinical characteristics (age, sex, weight for age and diagnosis on admission) of the children and background characteristics (age, sex and designation) of the assessors. Weight for age was calculated from WHO's z-score and categorised into not-underweight (weight for age score ≥-2 standard deviation), underweight (weight for age score is <-2 standard deviations), and severely underweight (weight for age score <-3 standard deviation)[39].

Obtaining a successful measurement of SpO_2_ was considered as the primary outcome of interest. At first, we presented the success rates for different cut off time points: within 20 seconds, within 30 seconds, within 40 seconds, within 50 seconds, within 60 seconds, within 90 seconds, within 120 seconds and within 180 seconds.

Performance time, defined by the time taken to obtain a successful measurement of SpO_2_, was considered the secondary outcome of interest. We checked the normality of the distribution of timing by device-related factors (type of pulse oximetry device), provider-related factors (age, sex and designation of assessor) and patient-related factors (age, sex, weight for age of the children, on-admission diagnosis/classification) using the Shapiro-Wilk test (Table S2 in the **Online Supplementary Document**) [40]. Since they were non-normally distributed, we presented the median estimates with an interquartile range through boxplots. We used the Wilcoxon Signed Rank test (for paired observations) to check the differences in performance time by type of pulse oximetry devices and the Ranksum test (for non-paired observations) to check the differences for other provider-related and patient-related factors. We also reported the median time taken by each assessor using radar plots. We used the Kruskal-Wallis test to assess whether there were any differences in the median time taken by each assessor.

The National IMCI Programme, in consultation with IMCI experts, set an a-priori cut-off of obtaining a successful measurement of SpO_2_ within 60 seconds as a feasible and acceptable performance time in a real-life setting. We used the Generalized Estimating Equation regression model to assess the effect of different device-related, provider-related, and patient-related factors on successful measurement of SpO_2_ within 60 seconds. The unadjusted and adjusted odds ratios were presented with 95% confidence intervals (CI). The model adequacy was assessed using Wald Statistics.

We used the prevalence-and bias-adjusted kappa (PABAK) statistics for reporting the agreement of identifying hypoxaemia (SpO_2_<90%) using Lifebox and Masimo devices, by various provider- and patient-related factors and by individual assessor [41]. The agreement levels were presented with point estimates (percentage) with 95% confidence intervals.

We have reported the percentage of people reporting very easy, easy, neither easy nor challenging, challenging and challenging for each statement using a bar graph.

We reported all differences or associations as significant at *P* < 0.05.

### Ethical considerations

Ethical approval for this study was obtained from the Institutional Review Board of icddr,b (PR-18054). We took informed and written consent from the primary caretaker/parents of the children and the assessors. We also obtained administrative approval from the Ministry of Health (IMCI Programme of Directorate General of Health Services) to conduct the study in Kushtia District Hospital.

## RESULTS

A total of 739 children were enrolled in the study. **Table 1** summarises the background characteristics of the children. Approximately 62% of the children were male, and around 52% were aged 2-11 months. Study nurses assessed around 61% of the children, and the remaining were assessed by the paramedics. Among the assessors, 14 (78%) were aged 25 years or below; seven (39%) were male, and 11 (61%) were females; 11 (61%) were nurses, and seven (39%) were paramedics. Table S3 in the **Online Supplementary Document** presents the number of pulse oximetry performed by each assessor.

**Table 1:** Background characteristics of the children hospitalised in Kushtia District Hospital and enrolled in the study (N = 739)

Characteristics	Category	n	%
Age	2-11 months	386	52
	12-59 months	353	48
Sex	Male	457	62
	Female	282	38
Weight for age	Not underweight	539	75
	Underweight (below -2SD)	102	14
	Severely underweight (below -3SD)	81	11
	Missing	17	
Diagnosis	Severe pneumonia or pneumonia	407	55
	Others	332	45
Type of assessor	Nurse	451	61
	Paramedic	288	39

SD – standard deviation

The assessors obtained a successful measurement of SpO_2_ in all attempts except one.

**Figure 1:**
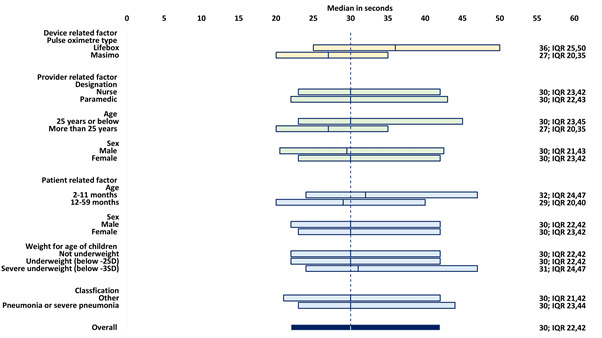
Performance time to obtain a successful measurement of SpO2, presented in median time taken (in seconds) by device-, assessor- and patient-related factors (N = 1478).

**Figure 1** presents the median performance time by various device-, provider-and patient-related factors. The median time taken to obtain a successful measurement was 30 seconds (IQR = 22, 42). The median time was significantly (Wilcoxon Signed Rank test for paired observations, *P* < 0.0001) higher among children who were assessed with a Lifebox device (36 seconds) than those who were assessed with a Masimo device (27 seconds). Similarly, the assessors aged more than 25 years required significantly (*P* < 0.0001) less time than assessors who were aged 25 years or less (median time 27 seconds vs 30 seconds). Moreover, the median time was more (*P* < 0.0001) among children aged 2-11 months (32 seconds) than that of children aged 12-59 months (29 seconds). We did not observe any significant difference (*P* = 0.296) between assessments conducted by nurses (median 30 seconds IQR = 23, 42) and paramedics (median 30 seconds IQR = 22, 43).

**Figure 2:**
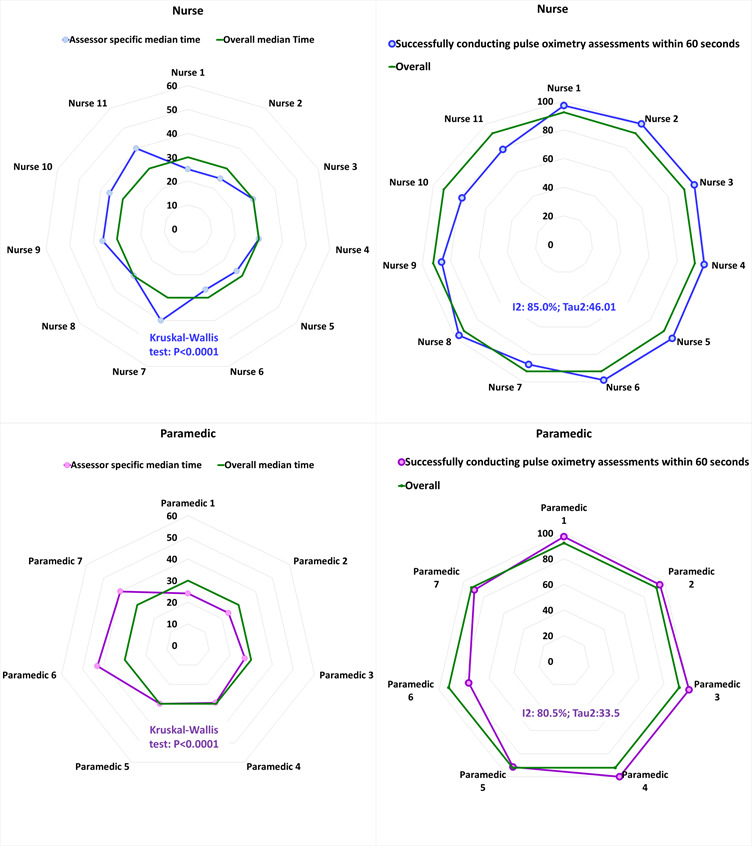
Performance time to obtain a successful measurement of SpO_2_ and proportion of successfully conducted pulse oximetry assessments within 60 seconds, presented in median time taken (in seconds) and in percentage (%) respectively, by individual assessors.

**Figure 2** illustrates the median performance time by each assessor and proportion of successfully conducted pulse oximetry assessments within 60 seconds. The median performance time was less than 60 seconds for all assessors. The median time taken by the nurses was 30 seconds which varied between 40 seconds (highest) and 25 seconds (lowest) among individual nurses (χ^2^ = 105.7, df = 10 df, *P* < 0.0001). Similarly, the median time taken by paramedics was 30 seconds which varied between 43 seconds (highest) and 24 seconds (lowest) among individual paramedics (χ^2^ = 105.6, df = 10, *P* < 0.0001).

The overall proportion of the successful assessments of pulse oximetry within 60 seconds is 92% for both nurses and paramedics. However, there exists variation on this proportion within the group of different assessors. Among the nurses and paramedics, the heterogeneity statistics I-squared are 85% and 80% and tau-squared are 46.01 and 33.5 respectively.

**Figure 3:**
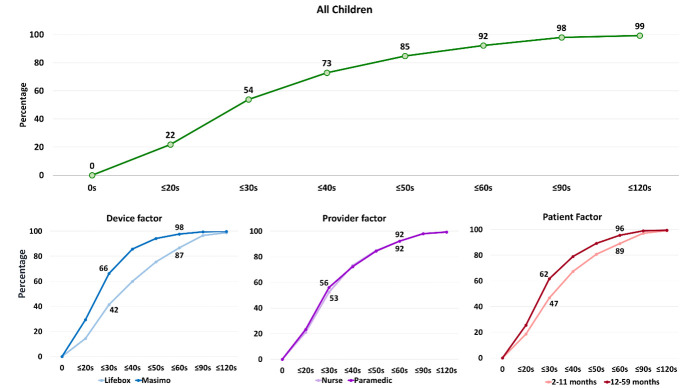
Rates of obtaining a successful measurement of SpO_2_, presented in percentage by different cut-off time points, and by pulse oximetry device type, assessor type and age of the children (N = 1478).

**Figure 3** presents the rates of obtaining a successful measurement at various cut-off time points by pulse oximetry device type, assessor type and age of the children. The assessors obtained a successful measurement within 30 seconds among 54% of children. It was 92% within 60 seconds and 99% within 120 seconds. The assessors obtained a successful measurement within 60 seconds among almost all children using a Masimo device, which was around 87% among children assessed with a Lifebox device. The assessors could successfully measure SpO_2_ within 60 seconds among more than 90% of children for both younger (2-11 months) and older (12-59 months) age groups.

**Figure 4:**
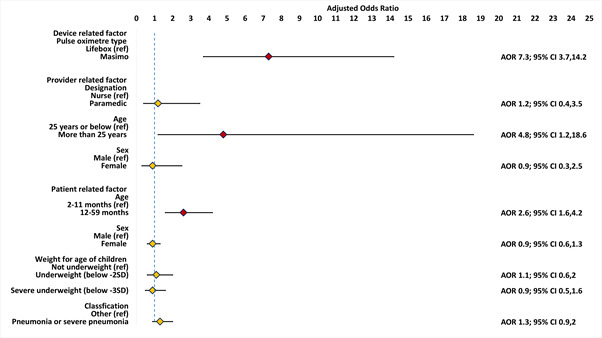
Associations between obtaining a successful measurement of SpO2 within 60 seconds and various device-, assessor- and patient-related factors, presented in adjusted odds ratio using a Generalised Estimating Equation (GEE) regression model (N = 1474).

**Figure 4** presents the effect of various device-, provider-, and patient-related factors on performance time to obtain a successful measurement of SpO_2_ within 60 seconds after controlling for covariates and confounders. The odds to obtaining a successful measurement of SpO_2_ within 60 seconds were 7.3 (95% CI = 3.7, 14.2, *P* < 0.0001) times higher when the assessments were conducted with a Masimo device than that of a Lifebox device. Similarly, assessors aged >25 years were 4.8 (95% CI = 1.2, 18.6, *P* = 0.03) times more likely to obtain a successful measurement of SpO_2_ within 60 seconds. Regarding patient-related factors, the odds to obtaining a successful measurement of SpO_2_ within 60 seconds were 2.6 (95% CI = 1.6, 4.2, *P* < 0.001) times higher among children aged 12-59 months than among children aged 2-11 months. The Wald Statistics 49.14 (*P* < 0.0001) obtained from the model demonstrated that the model was adequately fitted. Additional details are available in Table S4 in the **Online Supplementary Document**.

**Figure 5:**
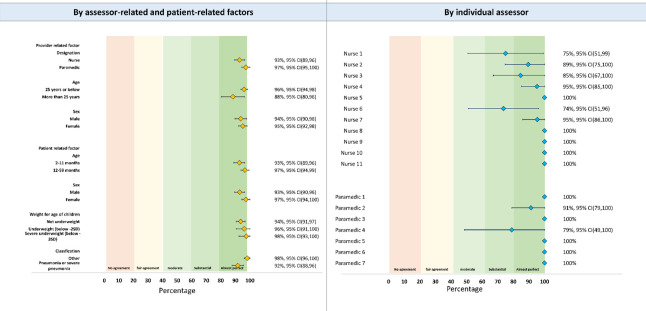
Agreement of identifying hypoxaemia using Lifebox and Masimo devices, presented in prevalence-adjusted and bias-adjusted kappa (PABAK) percentage with 95% CI by provider- and patient-related factors.

**Figure 5** presents the agreement of identifying hypoxaemia using Lifebox and Masimo devices, by provider- and patient-related factors and by individual assessor. We observed almost perfect level of agreement by various factors. The agreement levels were between moderate to almost perfect among assessors.

**Figure 6:**
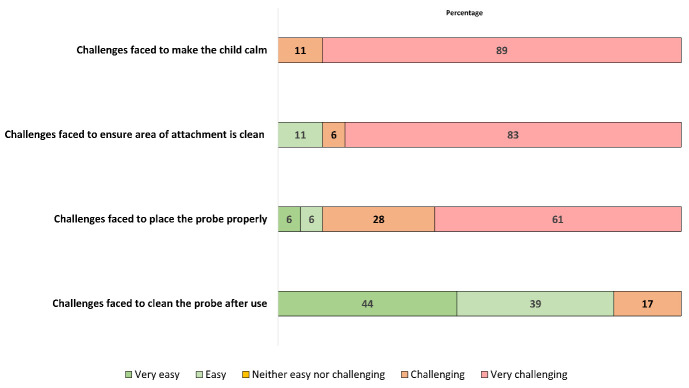
Feedback on challenges of performing pulse oximetry on children by assessors, presented as percentage of assessors reporting very easy, easy, neither easy or challenging, challenging and very challenging for each statement (N = 18).

**Figure 6** presents the result of the self-administered survey on the challenges of performing pulse oximetry on hospitalised children. All the assessors reported that they found it either very challenging or challenging to keep the baby was calm during performing pulse oximetry. Almost half of the assessors also reported that they found it very challenging to place the probe properly.

## DISCUSSION

The Government of Bangladesh decided to introduce pulse oximetry in routine IMCI services based on the WHO recommendation in 2014 and in-country consultation with IMCI experts and stakeholders [23]. Hence, the IMCI Programme of the Government of Bangladesh warranted this study to test the newly developed training module and measure the influence of various device-related, provider-related, and patient-related factors on pulse oximetry performance. Our study showed that the assessors could successfully use handheld pulse oximetry devices with relatively short training. The results of this study address some of the key evidence gaps related to the feasibility of adopting a short training and choice of pulse oximetry devices in the Bangladesh context.

We found that the minimally trained assessors obtained a successful measurement of SpO_2_ among more than 90% of the hospitalised children within 60 seconds. This is similar to findings reported by another study conducted in primary care clinics of rural Pakistan, which also employed study recruited personnel for pulse oximetry [42]. The results indicate that the study nurses and paramedics without previous experience of using a pulse oximetry device achieved necessary skills based on the short training module developed by the national committee. It is important considering the context of Bangladesh, where most of the IMCI service providers are nurses and paramedics and do not have any prior exposure to performing pulse oximetry in outpatient or emergency settings. The short training package is also programmatically more implementable and scalable in a resource-constrained high pneumonia-burden setting like Bangladesh [1,3,23]. Since more than 90% of the assessments were completed within 60 seconds, recommending pulse oximetry while assessing the respiratory rate for one minute will add minimum additional workload on the IMCI services providers even in high-volume facilities.

We compared the performance time taken by Masimo and Lifebox devices to obtain a successful measurement of SpO_2_. This information is crucial for the uptake of pulse oximetry in high-volume facilities. We found that the performance time was significantly shorter with a Masimo device. This finding is consistent with another study conducted by King et al., who found that Masimo and Lifebox devices' performance time was equivalent within ±7 seconds of one another [43]. Some studies also reported that the performance of Masimo devices was better than several other commercially available handheld pulse oximetry devices [28,30]. Masimo uses advanced Signal Extraction Technology (SET), which allows it to obtain a stable SpO_2_ reading faster even in challenging conditions such as patient movement and low perfusion [28,30]. The technical superiority of a Masimo device over most other handheld devices may be an important consideration for the Government of Bangladesh to select it for national scale-up, particularly in high-volume referral hospitals like district hospitals and sub-district hospitals. However, Masimo devices are almost twice as expensive as Lifebox devices [44]. Although the median performance time was more (9 seconds) with a Lifebox device, around 87% of the assessments were completed within one minute. In Bangladesh, the majority of the IMCI services are provided through the Union Health and Family Welfare Centres, which are primary care centres with a catchment population of around 25 000. Most of the Union Health and Family Welfare Centres are low-volume facilities and primarily deal with less severe cases. Considering the relative advantage of a Lifebox device over a Masimo device in price and the high rate of obtaining a successful measurement of SpO_2_ reading within 60 seconds, introducing Lifebox devices in Union Health and Family Welfare Centres and low volume sub-district hospitals can be a cost-effective investment for the Government of Bangladesh.

Nurses and paramedics provide the majority of the IMCI services in Bangladesh. Our study recruited nurses and paramedics as assessors to perform pulse oximetry. We did not observe any notable difference in their success rate or performance time. Therefore, the feasibility of conducting pulse oximetry by nurses and paramedics as routine IMCI service providers looks promising. Our study also suggested that older assessors were more likely to obtain a successful measurement of SpO_2_ within 60 seconds. This may be explained by the maturity and experience of the assessors, which are expected to gain with age. Younger and relatively inexperienced providers may require additional attention during initial training and post-training monitoring for standardising and ensuring quality. During training and monitoring, special attention should be given to ensure that the assessors are aware of the factors that might affect SpO_2_ readings, including anaemia, peripheral vasoconstriction, dark skin tone and skin discolouration of children [45].

Regarding patient-related factors, we found that obtaining a successful measurement of SpO_2_ among younger children takes significantly more time. The results are similar to a large multi-site study [29]. The health care workers interviewed in Bangladesh and Malawi also reported difficulties conducting pulse oximetry among smaller and younger children [35]. Pulse oximetry readings are significantly affected by patient movement and appropriateness of the probe size [28,30]. Hence, it is recommended to stabilise the child before and during the assessment. During pulse oximetry assessments, younger children requiring admission in the district hospital are expected to be more severe and less likely to be calm and stable. This can be one of the explanations for requiring additional time to obtain a successful measurement of SpO_2_ among younger children in our study. The other explanation is the design and appropriateness of the probe size. Although we used paediatric probes for both the devices, there could be issues with appropriate fit as around one-fifth of the children in rural Bangladesh are underweight, and one-third were stunted [46]. Hence, ensuring the availability of appropriate probe size is an important consideration for introducing pulse oximetry in routine IMCI services in Bangladesh and other LMICs with high burden of malnutrition.

Our study has several strengths and weaknesses. We included a reasonably large sample size ensuring 30-60 assessments per assessor, which allowed us to assess the pulse oximetry performance of each assessor separately. It was conducted in a district hospital. The results may not be nationally or regionally representative. However, Bangladesh is essentially a homogeneous country. There are minimal variations in the background characteristics and clinical profile of children admitted in secondary level public hospitals. We also acknowledge that we conducted the study among hospitalised children, who may not represent the children receiving IMCI services in busy outpatient settings. However, the number of irascible children among these hospitalised children are supposed to be higher than the children in outpatient [47]. Therefore, obtaining a successful measurement of SpO_2_ among relatively more severe hospitalised children is expected to be more difficult than children receiving IMCI services. Hence, the results of this study are still relevant for programme planning to introduce pulse oximetry in routine IMCI services. Furthermore, this study was conducted in a relatively controlled environment with study nurses and paramedics recruited and trained as part of a research study. Although we adopted the pulse oximetry training module developed by the national committee, our staff may have shown better performance than government-employed health service providers in routine IMCI services due to the higher level of motivation and enthusiasm [23,48]. Regarding data collection, we extracted information regarding the children's background characteristics and clinical information from routine hospital records. Therefore, the provision for presenting disaggregated estimates and adjusting for various comorbid conditions, such as specific diagnosis and spectrum of pathologies, while exploring the factors affecting the performance of pulse oximetry use was somewhat limited. Moreover, there can be issues with the validity of this information since documentation of paediatric inpatient care has always been a challenge in LMICs like Bangladesh [49]. Regarding analysis, performance time was non-normally distributed in our analysis (Figure S1 in the **Online Supplementary Document**). Hence, we had to conduct non-parametric tests, which are less efficient and robust than parametric tests. However, we tried to choose the most relevant and appropriate tests (eg, Signed Rank test, Ranksum test, Kruskal-Wallis test) based on the distribution of data and specific questions. Lastly, each assessor had two (Masimo and Lifebox) measurements of SpO_2_ on each child. Therefore, to account for the effect of intra-assessor correlation, we performed Generalised Estimating Equation regression to measure the effect of different patient-related, provider-related, and device-related factors on the time taken to obtain a stable SpO_2_ reading.

## CONCLUSIONS

Our study indicated that minimally trained assessors could obtain a successful measurement of SpO_2_ with handheld devices within 60 seconds in hospital inpatient settings with some effect of various device-, provider- and patient-related factors. The National IMCI Programme can use these findings for finalising the IMCI training modules and implementation package related to pulse oximetry. The feasibility and implementation challenges of introducing pulse oximetry in routine IMCI service needs to be further evaluated in real-life settings.

## Additional material


Online Supplementary Document
