# Supplementary material for: Success and time implications of SpO2 measurement through pulse oximetry among hospitalised children in rural Bangladesh: Variability by various device-, provider- and patient-related factors
Source: J Glob Health. 2022 Apr 23;12:04036. doi: 10.7189/jogh.12.04036 (PMC9041243; doi:10.7189/jogh.12.04036)
Supplement: Online Supplementary Document [file jogh-12-04036-s001.pdf]

Supplementary Figures:

Figure S1: Histograms of time-reading for obtaining a stable SpO_2 reading by groups to assess normality.

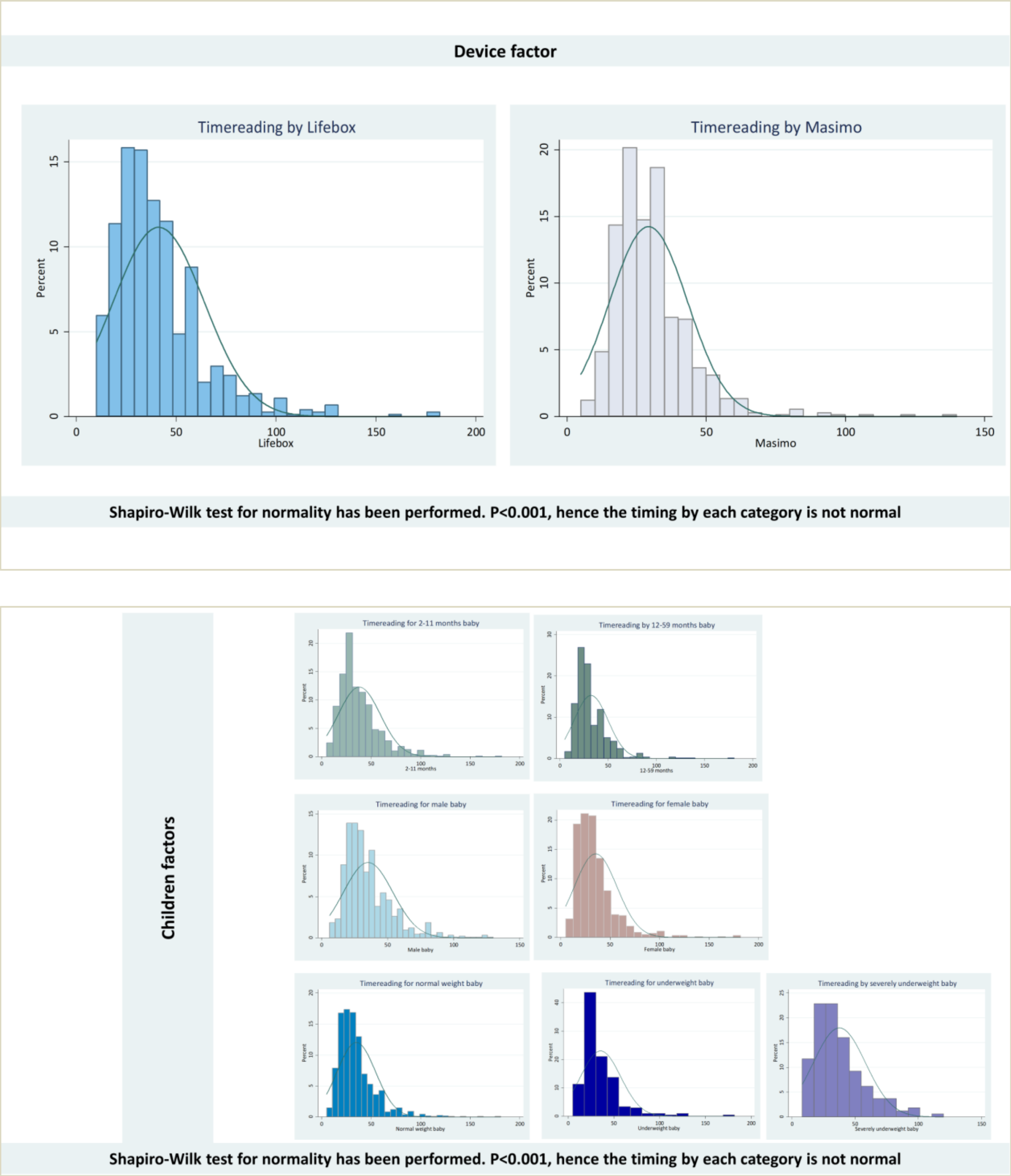

Assessor factors

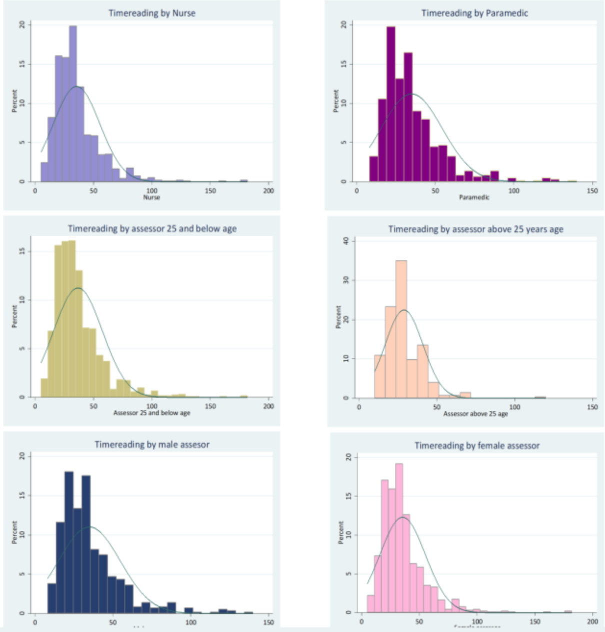

Shapiro-Wilk test for normality has been performed. P<0.001, hence the timing by each category is not normal

Diagnosis

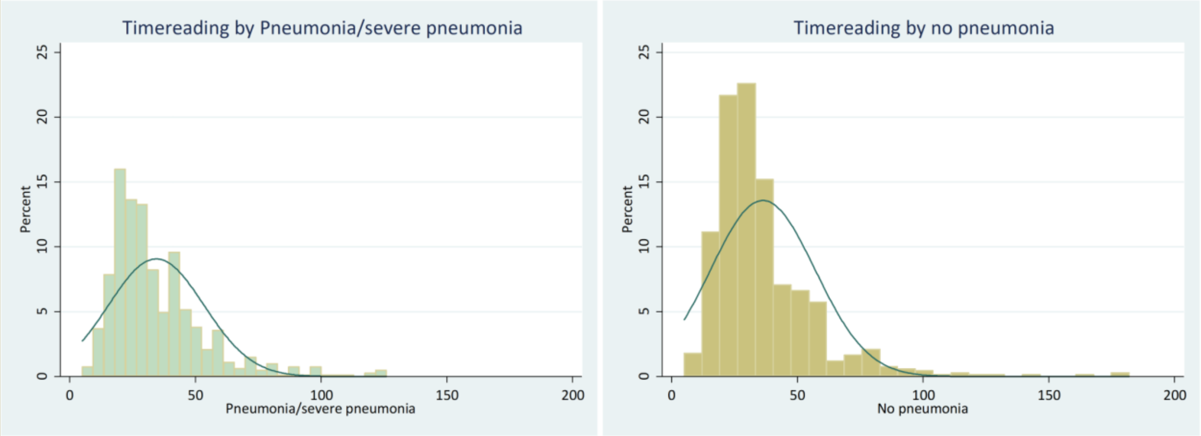

Shapiro-Wilk test for normality has been performed. P<0.001, hence the timing by each category is not normal

Supplementary Tables:

Table S1: Lifebox vs Masimo model, technical specification and price comparison.

Requirements	Lifebox A-care AH-M1	Masimo Rad-5v®
Price		
Price (USD)	200	500
General requirements		
For adults, children and neonates	Yes	Yes
For all skin pigmentations	No data found	No data found
Weight range for each patient category	No data found	No data found
SpO2 detection to include the range: 70–100%	Yes, 0-100%	Yes, 1-100%
SpO2 resolution: 1% or less.	1%	1%
SpO2 accuracy within ±3% for all patients and perfusion/movement conditions.	70%-100%: ± 2%; 0%-69%: not defined	2% with no motion low perfusion adults/paediatrics. 3% with motion and low perfusion neonates
Pulse rate range: 30–240 bpm.	25-250 bpm	25-240 bpm
Pulse rate resolution: 1 bpm or less	1 bpm	1 bpm
Pulse rate accuracy within ±3 bpm.	±2 bpm	3 bpm with no motion and in low perfusion, 5 bpm with motion
Audio		
Audible and visual alarms for low/high saturation and pulse rate, threshold set by user	Yes	Yes
Audible and visual alarms for sensor error or disconnected, system errors, low battery	Yes	Yes
Probes		
Capable of working with, and supplied with, adult, paediatric and neonatal reusable probes	Yes	Yes
Electrical characteristics		
Operated by replaceable battery power supply, either rechargeable or single use	Li-ion rechargeable battery or, 3 AA batteries	4XAA Alkaline batteries
Display		
%SpO_2	Yes	Yes
Pulse rate	Yes	Yes
Plethysmographic waveform	Yes	Yes
Alarm messages	Yes	Yes
Battery state indication	Yes	Yes
Others	No	Perfusion Index, Signal IQ (SIQ) bar
Optional		

Requirements	Lifebox A-care AH-M1	Masimo Rad-5v®
Internal data storage for patient trends and event log	Yes; 1-99 ID and 300 records for each ID	The Rad-5 can store 72 hours of SpO2 and Pulse Rate and Perfusion Index trend data, captured at 2 second intervals. This trend data can then be transferred to a PC for evaluation. Trend data is stored in non-volatile memory, so it is not erased when the device is shut off or when the batteries are replaced
FDA/CE Approval		
FDA/CE Approved	Yes	Yes,

Table S2: Medians of timing required to obtain a stable reading of SpO_2 by categories.

	**Min**	**Q1**	**Median**	**Q3**	**Max**	**Difference in median (in seconds)**	**Test of Choice for equality of distribution**	**P-value with more decimals for Wilcoxon test**
**All**	**5**	**22**	**30**	**42**	**182**			
Age of child								
2-11 months	5	24	32	47	182	3	Wilcoxon ranksum test	0.00000000426332543500000000000000000000<br>000000000000
12-59 months	5	20	29	40	181			
Sex								
Male	5	22	30	42	182	0	Wilcoxon ranksum test	0.50844932064946900000000000000000000000<br>000000000000
Female	6	23	30	42	130			
Weight for age of child								
Not Under-Weight (-2 SD and above)	5	22	30	42	181	Not Under-Weight and Under-Weight 0	Kruskal-Wallis	0.37635838777267500000000000000000000000<br>000000000000
Under-Weight (below -2SD)	5	22	30	42	182	Under-Weight and Severe Under-Weight 1		
Severely Under Weight (below -3SD)	8	24	31	47	120	Not Under-Weight and Severe Under-Weight 1		
Type of assessor								
Nurse	5	23	30	42	182	0	Wilcoxon ranksum test	0.29652587726540300000000000000000000000<br>000000000000
Paramedic	8	22	30	43	140			
Age of assessors								
25 and below	5	23	30	45	182	3	Wilcoxon ranksum test	0.00000001643088407600000000000000000000<br>000000000000
Above 25 years	10	20	27	35	120			
Sex of assessor								
Male	8	20.5	30	42.5	140	0	Wilcoxon ranksum test	0.15427529464996000000000000000000000000<br>000000000000
Female	5	23	30	42	182			
Type of PO								
Lifebox	10	25	36	50	182	9	Wilcoxon signrank test	0.00000000000000000000000000000000000000<br>000000001010
Masimo	5	20	27	35	140			
Diagnosis								
Pneumonia or severe pneumonia	5	21	30	42	126	0	Wilcoxon ranksum test	0.04413990347719940000000000000000000000<br>000000000000
Fever and other	5	23	30	44	182			

Table S3: Number of assessments by assessor type.

**Type of assessor**	**Number of assessments**
Nurse 1	64
Nurse 2	76
Nurse 3	78
Nurse 4	82
Nurse 5	74
Nurse 6	76
Nurse 7	86
Nurse 8	96
Nurse 9	80
Nurse 10	96
Nurse 11	94
Paramedic 1	104
Paramedic 2	92
Paramedic 3	76
Paramedic 4	38
Paramedic 5	94
Paramedic 6	88
Paramedic 7	84

Table S4: Generalised estimating equation (GEE) regression to assess the association between time taken to obtain successful measurement of SpO2 within 60 seconds and different characteristics of participants presented in adjusted odds ratio and 95% Confidence Interval (N=1474).

	%	Crude Odds ratio	95% CI Lower limit	95% CI Upper limit	P value	Adjusted Odds ratio	95% CI Lower limit	95% CI Upper limit	P value
**Diagnosis**									
Fever and other	92								
Pneumonia or severe pneumonia	93	1.13	0.76	1.67	0.55	1.33	0.87	2.04	0.19
**Age of child**									
2-11 months	89								
12- 59 months	96	2.07	1.32	3.24	0.00	2.64	1.64	4.24	0.00
**Sex of child**									
Male	92								
Female	92	0.92	0.63	1.36	0.69	0.87	0.58	1.32	0.52
**Weight for age of child**									
Not Under-Weight (-2 SD and above)	93								
Under-Weight (below -2SD)	92	1.06	0.60	1.86	0.84	1.09	0.60	1.98	0.78
Severely Under Weight (below -3SD)	89	0.87	0.49	1.57	0.65	0.87	0.48	1.57	0.64
**Type of pulse oximetry**									
Lifebox	87								
Masimo	98	6.97	3.41	14.25	0.00	7.29	3.74	14.20	0.00
**Type of assessors**									
Nurse	92								
Paramedic	92	0.98	0.31	3.07	0.98	1.19	0.41	3.44	0.75
**Age of assessors (in years)**									
25 years and below	91								
Above 25	98	5.86	0.63	54.46	0.12	4.75	1.21	18.58	0.03
**Sex of assessor**									
Male	92								
Female	92	1.06	0.34	3.32	0.91	0.85	0.30	2.45	0.76
